# PPAR*γ*: The Portrait of a Target Ally to Cancer Chemopreventive Agents

**DOI:** 10.1155/2008/436489

**Published:** 2008-09-03

**Authors:** Ioannis Sainis, Katerina Vareli, Vasilios Karavasilis, Evangelos Briasoulis

**Affiliations:** ^1^Department of Biological Applications and Technologies, University of Ioannina, Ioannina 45110, Greece; ^2^Institute of Cancer Research, Royal Cancer Hospital, Sutton Surrey, London SM2 5NG, UK; ^3^Department of Medical Oncology, School of Medicine, University of Ioannina, Ioannina 45110, Greece

## Abstract

Peroxisome proliferator-activated receptor-gamma (PPAR*γ*), one of three ligand-activated transcription factors named PPAR, has been identified as a molecular target for cancer chemopreventive agents. PPAR*γ* was initially understood as a regulator of adipocyte differentiation and glucose homeostasis while later on, it became evident that it is also involved in cell differentiation, apoptosis, and angiogenesis, biological processes which are deregulated in cancer. It is now established that PPAR*γ* ligands can induce cell differentiation and yield early antineoplastic effects in several tumor types. Moreover, several bioactive natural products with cancer protecting potential are shown to operate through activation of PPAR*γ*. Overall, PPAR*γ* appears to be a prevalent target ally to cancer chemopreventive agents and therefore pursuing research in this area is of great relevance.

## 1. INTRODUCTION

Peroxisome proliferator-activated receptors (PPARs) are ligand-activated nuclear receptors that function as transcription factors regulating the expression of genes involved in lipid biosynthesis, glucose metabolism, as well as cell proliferation, differentiation, and survival [1–4]. Their discovery was driven by search of a molecular target for peroxisome proliferators, a group of agents named after their property to increase peroxisomes in rodent liver [5, 6]. Later on, activity studies helped elucidate the versatile role of these molecules in modulating diverse biological functions such as metabolism, tissue remodeling, inflammation, angiogenesis, and carcinogenesis [7–11]. Three PPAR gene types have been identified: *α*, *β*/*δ*, and *γ* [12, 13]. Between them, PPAR*γ* is the most intensively investigated [14, 15].

## 2. THE HUMAN PPAR*γ* GENE

The human PPAR*γ* gene consists of six coding exons located at chromosome 3p25.2 and extends approximately over 100 kb of genomic DNA [16]. Three major transcriptional start sites have identified where three mature mRNAs originate from, differing in their 5′ untranslated regions [17, 18]. Notably PPAR*γ*1 and PPAR*γ*3 mRNAs code for the same protein of 475 amino acids, while PPAR*γ*2 transcript codes for a different protein which contains an additional 28 N-terminal amino acids [19].

### 2.1. Tissue distribution of different PPAR*γ* isoforms

The PPAR*γ*1 is found in virtually all tissues, such as liver, skeletal muscle, prostate, kidney, breast, intestine, and the gonads. The PPAR*γ*2 is the major PPAR*γ* isoform expressed mainly in adipose tissue where it normally operates as an adipocyte-specific transcription factor in preadipocytes and regulates adipose tissue differentiation, and the PPAR*γ*3 isoform is restricted to adipose tissue and large intestine [18, 20],

### 2.2. PPAR*γ* protein structure and function

**Figure 1:**
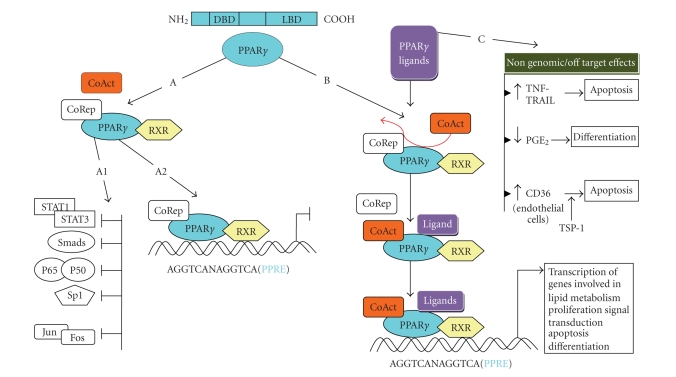
*Peroxisome proliferator-activated receptor-*γ* and ligands: pathways and functions.* PPAR*γ* protein exhibits a structural organization consisting of three functional domains: an N-terminal domain, a DNA-binding domain (DBD) and a carboxy-terminal ligand binding domain (LBD). PPAR*γ* forms heterodimers with a second member of the nuclear receptor family, the retinoic X receptor (RXR). Unliganded PPAR*γ* suppresses transcription (pathway A) either by interfering with key transcription factors (pathway A1) or through recruitment of corepressors (CoRep) on a PPRE element (pathway A2). Ligand binding to PPAR*γ* (pathway B) triggers conformational changes that lead to dissociation of corepressors (CoRep) and subsequent association of coactivators (CoAct). The complex is binding to PPREs and triggers transcription (pathway B). PPARs ligands can also exert their action through PPAR*γ*-independent mechanisms also (pathway C). For instance in NSCLC cell lines activation of TNF-TRAIL induce apoptosis, while PGE_2_ degradation, trough 15-hydroxyprostagladin dehydrogenase induction, results in enhanced epithelial differentiation. In endothelial cells PPAR*γ* ligands can markedly boost expression of CD36 which functions as the receptor of endogenous antiangiogenic molecule thrombospondin-1, thereby potentiating the apoptotic response. (PFAs: polyunsaturated fatty acids, TZDs: thiazolidinediones, PPRE: peroxisome proliferator response element, TNF: tumor necrosis factor, TRAIL: TNF-related apoptosis-inducing ligand, NSCLC: non-small cell lung carcinoma).

Similar to other members of the nuclear hormone receptors superfamily, PPAR*γ* protein has three functional domains: the N-terminal domain, the DNA-binding domain, and a carboxy-terminal ligand-binding pocket (Figure 1).

PPAR*γ* protein receptor is activated by a number of endogenous and exogenous ligands of various potencies. Among pharmaceutical compounds, thiazolidinedione (TZD) class of insulin-sensitizing drugs (also called glitazones) are best known to operate as ligands to PPAR*γ* [21, 22] while long-chain polyunsaturated fatty acids are the most well-characterized endogenous ligands [23].

The activated PPAR*γ* protein becomes operational following its heterodimerization with retinoid X receptors (RXR) [24]. The PPAR*γ*/RXR complex translocates to the nucleus where it binds to target genes which contain a peroxisome proliferator response element (PPRE). A PPRE consist of a direct repetition of the consensus sequence AGGTCA separated by a single nucleotide (Direct repetition; DR1) [17]. To initiate transcriptional regulation of PPRE-bearing genes, the PPAR*γ*/RXR complex requires accessory proteins to bind on. These proteins can either trigger (coactivators) or represses gene transcription (corepressors) (Figure 1). It must be noted though that besides their PPAR*γ*-dependent genomic effects, PPAR*γ* ligands can also influence cellular biology via nongenomic, PPAR*γ*-independent events [25] (Figure 1).

As a rule, the transcriptional activity of PPAR*γ* is negatively modulated through phosphorylation by MAPK [26–28]. Phosphorylation of human PPAR*γ*1 protein at Ser-84 site restrains its function [27], and phosphorylation of PPAR*γ*2 modifies the A/B domain and reduces its ligand binding affinity [29]. However, not all phosphorylation events are inhibitory. For example, it has been found that missense mutation which results in the conversion of proline to glutamine at position 115 can render PPAR*γ*2 constitutively active through modulation of the MAPK-dependent phosphorylation status of serine 114 [30] while phosphorylation by protein kinase A (PKA) was shown to enhance its activity [31].

Until now, three molecular processes have been proposed for the termination and downregulation of PPAR*γ* signaling: the phosphorylation of Ser-84/112 of PPAR*γ*1/2 by ERKs [27], the proteasomal degradation of ligand-activated PPAR*γ* [32], and the interaction with MEKs, which promotes its expulsion from the nucleus [33].

## 3. PPAR*γ* IN CANCER

Early studies portrayed PPAR*γ* as an important regulator of preadipocyte differentiation and glucose homeostasis. Later on, it was identified that PPAR*γ* regulates biological processes which are considered hallmarks of cancer such as cell differentiation, apoptosis, and angiogenesis. This knowledge, coupled with data showing that PPAR*γ* ligands could yield anticancer effects in several cell types, led researches postulate a role for PPAR*γ* in carcinogenesis [11, 34, 35].

Apoptosis is believed to be a fundamental molecular mechanism through which PPAR*γ* activators exert their action against cells which undergo malignant transformation [36–38]. Moreover, apart from their direct inhibitory effects on cancerous transformed cells, PPAR*γ* can also inhibit angiogenesis which is a prerequisite for tumor formation and growth [39–41]. It is suggested that the antiangiogenic activity of PPAR*γ* can be accomplished either by blocking the production the angiogenic ELR+CXC chemokines by cancer transformed cells or by inducing expression of the thrombospondin-1 receptor CD36 in endothelial cells [42–44] In addition, latest exciting data, which showed that PPAR*γ* agonists were able to inhibit the canonical WNT signaling in human colonic epithelium, raises hopes that such agents can possibly block cancer initiation at a stem cell level [45].

It must be underlined herein that despite demonstration of cancer-preventive effects of PPAR*γ* ligands in vitro, clinical trials and animal models failed so far to show significant benefits [46]. The fact that PPAR*γ* ligands have been used in clinic trials at concentrations above those needed to elicit receptor agonistic activity poses questions for receptor-independent off-target effects [47].

### 3.1. PPAR*γ* and gastrointestinal cancer

PPAR*γ* are heterogeneously expressed throughout the gastrointestinal epithelium, showing significant differences in abundance, distribution, and functions. This protein is principally expressed in differentiated epithelial colonic cells, preferably in the proximal colon [48]. Sarraf et al. showed that PPAR*γ* activation could stimulate a program that is characteristic of colonic cell differentiation [49].

A functional genomics analysis conducted for the identification of PPAR*γ* gene targets revealed that the majority of these genes were transcribed throughout the colon, but their expression varied in cells purified from the proximal colon and in those from the distal colon. Metabolic functions of PPAR*γ* were elicited primarily in the proximal colon, whereas signaling functions were recognized in the distal colon. Interestingly, TZDs transactivated the PPAR*γ* gene targets at the proximal colon but repressed them in the distal colon. TSC22, a TGF*β* target gene known to inhibit colon cell proliferation, was also identified as a PPAR*γ* target gene [50]. It is worth mentioning that both TGF*β* and PPAR*γ* pathways attenuate during transition from adenoma to carcinoma [51]. From a pharmacological point of view, Yamazaki at al. showed that activation of the RXR/PPAR*γ* heterodimer by their respective ligands could be considered a useful chemopreventive strategy for colorectal cancer. They found that a combination of the RXR alpha ligand 9-cis-retinoic acid with ciglitazone synergistically inhibited the cell growth and induced apoptosis in Caco2 human colon cancer cells that expressed high levels of p-RXR alpha protein [52].

In the most widely used preclinical model of sporadic colon carcinogenesis, the azoxymethane-treated mice, activation of PPAR*γ* suppressed carcinogenesis but only before damage to the APC/beta-catenin pathway [53]. However, two papers published ten years ago reported that troglitazone and rosiglitazone increased occurrence of colon tumors in mice-caring mutations in the *APC* gene [48, 54]. Moreover, although pioglitazone was later reported to suppresses colon tumor growth in Apc+/− mice [55], biallelic knockdown of PPAR*γ* in colonic epithelial cells was associated with an increase of tumor incidence [56]. It should be reminded, however, that although TZDs are considered pure PPAR agonists, they also wield off-target effects not mediated through linkage to PPAR receptors. An in-depth analysis of the role of TZDs against colon cancer can be facilitated through development of tissue-specific PPAR*γ* knockout mice [57]. Interestingly, a small phase II clinical trial using troglitazone failed to document tumor responses in patients with advance stage metastatic colon cancer [58].

Overall, existing evidence indicates that PPAR*γ* agonists have a potential to inhibit cancer formation in the distal colon, but they are practically inactive in advanced stages of colon cancer.

### 3.2. PPAR*γ* and lung cancer

Lung cancer is a major global health problem because of its incidence and mortality. It remains the top cancer killer worldwide to which early-detection strategies and development of new therapies failed so far to improve its lethal outcome [59]. This tobacco-related cancer epidemic persists despite public implementation of tobacco control measures because the majority of tobacco-smoke users declare powerlessness to quit. Therefore, the search for potent chemopreventive agents and the development of effective chemoprevention strategies for lung cancer is a viable pursuit highly justified [60, 61].

Several studies have shown that PPAR*γ* agonists can inhibit growth and induce changes associated with differentiation and apoptosis in lung cancer [62–64]. TZDs induced upregulation of PTEN and p21, downregulation of cyclins D and E, and reduced expression of fibronectin and its receptor integrin *α*5*β*1 in human lung carcinoma cell lines [65–68].

A first evidence of clinical efficacy of PPAR*γ* agonists as cancer chemopreventives in lung cancer was recently published. A retrospective analysis of a database from ten Veteran Affairs medical centers revealed a significant reduction (33%) in lung cancer risk in diabetic patients who were treated with TZDs compared with nonusers of TZDs [69]. However, other studies damped early this enthusiasm by showing that diabetic patients treated with TZDs were at increased risk for cardiovascular complications [70].

It is critical to understand that cancer-protecting effects of PPAR*γ* agonists in lung cancer can be PPAR*γ* dependent but also PPAR*γ* independent [71]. Characteristically, TZDs suppressed the expression of antiapoptotic mediator prostaglandin E(2) in NCLC cells through induction of 15-hydroxyprostagladin dehydrogenase [72] and enhanced TRAIL-induced apoptosis through upregulation of death receptor 5 DR5 and downregulation of c-FLIP in human lung cancer cells [73].

The combination of PPAR*γ* agonists with other chemopreventive agents emerges as a challenging issue in lung cancer chemoprophylaxis. Notably, an amazing synergy of clinically achievable concentrations of lovastatin (an HMG-CoA reductase inhibitor) and troglitazone was recently shown against lung cancer cells [74]. This effect was accompanied by synergistic modulation of E2F-1, p27∧Kip1, CDK2, cyclin A and RB. In another study, a combination of low-doses of MK886 (5-lipoxygenase activating protein-directed inhibitor), ciglitazone and 13-cis-retinoic acid, also demonstrated synergistic inhibitory activity against lung cancer cells [75]. These studies provide a framework for the development of rationally designed drug combinations aimed to target simultaneously the PPAR*γ* and other cofactors.

### 3.3. PPAR*γ* and other malignancies

Epidemiological studies suggested that high consumption of carotenoids (known PPAR*γ* activators) could protect women from the development of breast cancer [76, 77]. These findings are also supported by experiments which show that activation of PPAR*γ* can induce terminal differentiation, cell cycle arrest, or apoptosis of preneoplastic and cancerous mammary epithelial cells [78–80]. Unfortunately, this is not the case for advanced breast cancer: a phase II trial of troglitazone in patients with breast cancer metastases failed recently to prove clinical benefits [81].

Prostate cancer appears to be an attractive tumor target for PPAR*γ* agonists because cancerous prostate cells express higher levels of PPAR*γ* compared with their normal counterparts [82]. Moreover, it has been shown that PPAR*γ*1/2 activation suppressed the high level of endogenous COX-2 in normal prostate epithelial cells [83] while TZDs mediated apoptosis in prostate cancer cells through inhibition of Bcl-xL/Bcl-2 functions [84]. In the clinical setting, reduction and prolonged stabilization of prostate-specific antigen levels were demonstrated in patients treated with troglitazone [82, 85]. The above data provide a rationale to consider investigating PPAR*γ* ligands for their role in preventive and possibly therapeutic management of prostate cancer.

In gynecological cancer, Wu et al. reported that rosiglitazone could block or delay the development of hyperplasia and subsequent endometrial cancer. This PPAR*γ* agonist induced apoptosis in both PTEN intact and PTEN null cancer cell lines and decreased proliferation of the endometrial hyperplastic lesions in a PTEN(+/−) murine model [86].

In human pancreatic cancer cell lines, treatment with TZDs was found to induce cell cycle arrest and increase expression of pancreatic differentiation markers [87, 88]. Moreover, activation of PPAR*γ* together with RXR resulted in suppression of pancreatic cancer cell growth through suppression of cyclin D1 [89].

Among sarcoma tumors, it is liposarcomas which are considered targets for PPAR*γ* agonists because they show a high expression of this nuclear receptor [90]. However, although pioglitazone was found capable to terminally differentiate human liposarcoma cells in vitro, it failed an early phase II trial despite induced changes in relevant target genes [91].

In thyroid cancer, a functional chromosomal translocation of part of *PAX*8 gene which encodes the DNA-binding domain to the activation domain of the PPAR*γ* gene has been detected in patients with follicular type carcinoma [92]. This chimeric fusion protein is resistant to PPAR*γ* ligands, invalidating any anticancer effects of PPAR*γ* ligands in this setting. However, it has been suggested that PPAR*γ* ligands could have activity in combination with retinoids and/or histone deacetylase inhibitors in thyroid tumors which express both PPAR*γ* and also RXR*γ* [93, 94].

## 4. PPAR*γ* AS A MEDIATOR TO CANCER PROTECTING NATURAL PRODUCTS

Evidence has accumulated which affirms that bioactive natural compounds can play an important role in cancer chemoprevention through modulation of PPAR*γ*. Preclinical studies and epidemiological data support that tumor growth and metastasis can be restrained or delayed by several herbal products [95–98]. Moreover, it is believed that novel agents derived from bioactive phytochemicals can be used as adjuncts to enhance therapeutic efficacy of standard treatments [99, 100]. Among natural products, triterpenoids, flavononoids, carotenoids, and linoleic acid are the most extensively studied as cancer chemopreventives and have invariably been found to operate as PPAR*γ* activators.

Terpenoids of plant origin have shown antitumor activity which indicates a potential role for these compounds as cancer chemopreventives [100–102]. Specifically, 2-cyano-3,12-dioxooleana-1,9-dien-28-oic acid (CDDO), a synthetic triterpenoid, which was shown to activate PPAR*γ* and induce growth arrest and apoptosis in treated breast cancer cells [103]; also, glycyrrhizin the major triterpene gycoside phytochemical in licorice root and the triterpenoid acid betulinic acid which is found in the bark of several species of plants, both have shown pro-PPAR*γ* activities in cancer cells. These phytochemicals were found to induce expression of proapoptotic protein caveolin-1 and the tumor-suppressor gene Kruppel-like factor-4 (KLF-4) in colon and pancreatic cancer cells [104, 105]. It should though be noted that although caveolin-1 is generally considered a proapoptotic molecule, it has also been associated with drug resistance and possibly metastasis [106]. It is believed that some PPAR-*γ* agonists induce whilst others repress caveolin-1 [107].

Isoflavones are well known to function as phytoestrogens. They bind to the estrogen-related receptors but also to PPAR*α* and PPAR*γ* [108]. As a result, their biological effects are determined by the balance between activated ERs and PPAR*γ* [109]. Liang et al. investigated apigenin, chrysin, and kaempferol in mouse macrophages and found that these flavonoids stimulated PPAR*γ* transcriptional activities as allosteric effectors rather than pure agonists [110]. In the clinical setting, purified isoflavones have only been investigated for safety, bioavailability, and pharmacokinetics in men with early-stage prostate cancer [111–114].

Carotenoids are another class of phytochemicals found to activate PPAR**γ** in cancer cells. Hosokawa et al. reported that the edible carotenoid fucoxanthin, when combined with troglitazone, induced apoptosis of Caco-2 cells [115]. Moreover, in epidemiological studies, consumption of carotenoids was shown to protect against breast cancer [76, 77]. Interestingly, Cui et al. unveiled recently the molecular mechanisms which underlie the chemopreventive activity of *β*-carotene against breast cancer. They found that *β*-carotene significantly increased PPAR*γ* mRNA and protein levels in a time-dependent fashion, while 2-chloro-5-nitro-N-phenylbenzamide (GW9662), an irreversible PPAR*γ* antagonist, attenuated apoptosis caused by *β*-carotene in cancer-transformed cells [36].

Linoleic acid, a naturally occurring omega-6 fatty acid which is abundant in many vegetable oils, has been studied comprehensively for its prophylactic effects against cancer formation [116]. Conjugated linoleic acid, which is found especially in eggs and in the meat and dairy products of grass-fed ruminants, was shown to modulate cell-cell adhesion and invasiveness of MCF-7 cells through regulation of PPAR*γ* expression [117]. Moreover *α*-eleostearic acid (ESA), a linolenic acid isomer, induced apoptosis in endothelial cells and inhibited angiogenesis, also through activation of PPAR*γ* [118]. More recent studies brought up additional evidence and provided insights into molecular mechanisms of the protective effects of linoleic acid against colon cancer. Yasui et al. reported that 9trans-11trans-conjugated linoleic acid inhibited the development of azoxymethane-induced colonic aberrant crypt foci in rats at preinitiation and postinitiation level through activation of PPAR*γ* and downregulation of cyclooxygenase-2 and cyclin D1 [119]. In addition, Sasaki at al. showed that linoleic acid was capable to inhibit azoxymethane-induced transformation of intestinal cells and tumor formation [120]. In most studies, the differentiation-promoting and carcinogenesis-blocking effects were mostly attributed to activation of PPAR*γ* by linoleic acid products [121]. Finally, apart from its direct action as a PPAR*γ* activator, linoleic acid was found to modulate interactions between PPAR*β*/*δ* and PPAR*γ* isoforms [122].

Finally, in the class of capsaicinoids, capsaicin, the spicy component of hot peppers, was shown to induce apoptosis of melanoma as well as colon and prostate cancer cells, and was associated with activation of the PPAR*γ* in the case of colon cancer [123–125]. However, controversy exists regarding cancer-preventing and cancer-promoting effects of capsaicin [126, 127].

It must be noted that besides their PPAR*γ*-mediated effects, natural products can also induce transcription of detoxification enzymes glutathione S-transferases (GST) which are known to protect cells from chemical-induced carcinogenesis [128, 129]. Recently, Park et al. examined GSTA2 gene induction by thiazolidinedione and 9-cis-retinoic acid and investigated the molecular basis of PPAR*γ*/RXR-mediated GSTA2 induction in the H4IIE hepatocytes. They found that both PPAR*γ* and RXR agonists could increase the expression of GSTA2 but treatment of cells with a combination of PPAR*γ* and RXR agonists produced synergistic increase [130]. This data suggest that cancer-preventive functions of PPAR*γ* activators may be related to some extent to a parallel induction of GSTA2.

## 5. CONCLUSION

Existing data suggest that peroxisome proliferator-activated receptor-gamma (PPAR*γ*) is a potential target ally to cancer chemopreventive agents. Although PPAR*γ* was first understood as a key regulator of adipocyte differentiation and glucose homeostasis, it is now recognized that it is also involved in cell proliferation, differentiation, apoptosis, and angiogenesis. Meticulous research for PPAR*γ* agonists with potency to function as cancer chemopreventive agents is highly warranted.

## References

[1] Feige JN, Gelman L, Michalik L, Desvergne B, Wahli W (2006). From molecular action to physiological outputs: peroxisome proliferator-activated receptors are nuclear receptors at the crossroads of key cellular functions. *Progress in Lipid Research*.

[2] Dreyer C, Krey G, Keller H, Givel F, Helftenbein G, Wahli W (1992). Control of the peroxisomal *β*-oxidation pathway by a novel family of nuclear hormone receptors. *Cell*.

[3] Desvergne B, Wahli W (1999). Peroxisome proliferator-activated receptors: nuclear control of metabolism. *Endocrine Reviews*.

[4] Chawta A, Repa JJ, Evans RM, Mangelsdorf DJ (2001). Nuclear receptors and lipid physiology: opening the X-files. *Science*.

[5] Issemann I, Green S (1990). Activation of a member of the steroid hormone receptor superfamily by peroxisome proliferators. *Nature*.

[6] Lock EA, Mitchell AM, Elcombe CR (1989). Biochemical mechanisms of induction of hepatic peroxisome proliferation. *Annual Review of Pharmacology and Toxicology*.

[7] Kersten S, Desvergne B, Wahli W (2000). Roles of PPARS in health and disease. *Nature*.

[8] Corton JC, Anderson SP, Stauber A (2000). Central role of peroxisome proliferator-activated receptors in the actions of peroxisome proliferators. *Annual Review of Pharmacology and Toxicology*.

[9] Panigrahy D, Singer S, Shen LQ (2002). PPAR*γ* ligands inhibit primary tumor growth and metastasis by inhibiting angiogenesis. *The Journal of Clinical Investigation*.

[10] Nicol CJ, Yoon M, Ward JM (2004). PPAR*γ* influences susceptibility to DMBA-induced mammary, ovarian and skin carcinogenesis. *Carcinogenesis*.

[11] Martinasso G, Oraldi M, Trombetta A (2007). Involvement of PPARs in cell proliferation and apoptosis in human colon cancer specimens and in normal and cancer cell lines. *PPAR Research*.

[12] Hertz R, Bar-Tana J (1998). Peroxisome proliferator-activated receptor (PPAR) alpha activation and its consequences in humans. *Toxicology Letters*.

[13] Larsen LK, Amri E-Z, Mandrup S, Pacot C, Kristiansen K (2002). Genomic organization of the mouse peroxisome proliferator-activated receptor *β*/*δ* gene: alternative promoter usage and splicing yield transcripts exhibiting differential translational efficiency. *Biochemical Journal*.

[14] Berger J, Moller DE (2002). The mechanisms of action of PPARs. *Annual Review of Medicine*.

[15] Lehrke M, Lazar MA (2005). The many faces of PPAR*γ*. *Cell*.

[16] Beamer BA, Negri C, Yen C-J (1997). Chromosomal localization and partial genomic structure of the human peroxisome proliferator activated receptor-gamma (hPPAR*γ*) gene. *Biochemical and Biophysical Research Communications*.

[17] Fajas L, Auboeuf D, Raspé E (1997). The organization, promoter analysis, and expression of the human PPAR*γ* gene. *The Journal of Biological Chemistry*.

[18] Fajas L, Fruchart J-C, Auwerx J (1998). PPAR*γ*3 mRNA: a distinct PPAR*γ* mRNA subtype transcribed from an independent promoter. *FEBS Letters*.

[19] Tontonoz P, Hu E, Graves RA, Budavari AI, Spiegelman BM (1994). mPPAR*γ*2: tissue-specific regulator of an adipocyte enhancer. *Genes & Development*.

[20] Mukherjee R, Jow L, Croston GE, Paterniti JR (1997). Identification, characterization, and tissue distribution of human peroxisome proliferator-activated receptor (PPAR) isoforms PPAR*γ*2 versus PPAR*γ*1 and activation with retinoid X receptor agonists and antagonists. *The Journal of Biological Chemistry*.

[21] Nolte RT, Wisely GB, Westin S (1998). Ligand binding and co-activator assembly of the peroxisome proliferator- activated receptor-*γ*. *Nature*.

[22] Uppenberg J, Svensson C, Jaki M, Bertilsson G, Jendeberg L, Berkenstam A (1998). Crystal structure of the ligand binding domain of the human nuclear receptor PPAR*γ*. *The Journal of Biological Chemistry*.

[23] Issemann I, Prince RA, Tugwood JD, Green S (1993). The peroxisome proliferator-activated receptor: retinoid X receptor heterodimer is activated by fatty acids and fibrate hypolipidaemic drugs. *The Journal of Molecular Endocrinology*.

[24] Kliewer SA, Umesono K, Noonan DJ, Heyman RA, Evans RM (1992). Convergence of 9-*cis* retinoic acid and peroxisome proliferator signalling pathways through heterodimer formation of their receptors. *Nature*.

[25] Burgermeister E, Seger R (2007). MAPK kinases as nucleo-cytoplasmic shuttles for PPAR*γ*. *Cell Cycle*.

[26] Hu E, Kim JB, Sarraf P, Spiegelman BM (1996). Inhibition of adipogenesis through MAP kinase-mediated phosphorylation of PPAR*γ*. *Science*.

[27] Adams M, Reginato MJ, Shao D, Lazar MA, Chatterjee VK (1997). Transcriptional activation by peroxisome proliferator-activated receptor *γ* is inhibited by phosphorylation at a consensus mitogen-activated protein kinase site. *The Journal of Biological Chemistry*.

[28] Burns KA, Vanden Heuvel JP (2007). Modulation of PPAR activity via phosphorylation. *Biochimica et Biophysica Acta*.

[29] Shao D, Rangwala SM, Bailey ST, Krakow SL, Reginato MJ, Lazar MA (1998). Interdomain communication regulating ligand binding by PPAR-*γ*. *Nature*.

[30] Ristow M, Müller-Wieland D, Pfeiffer A, Krone W, Kahn CR (1998). Obesity associated with a mutation in a genetic regulator of adipocyte differentiation. *The New England Journal of Medicine*.

[31] Lazennec G, Canaple L, Saugy D, Wahli W (2000). Activation of peroxisome proliferator-activated receptors (PPARs) by their ligands and protein kinase A activators. *Molecular Endocrinology*.

[32] Floyd ZE, Stephens JM (2002). Interferon-*γ*-mediated activation and ubiquitin-proteasome-dependent degradation of PPAR*γ* in adipocytes. *The Journal of Biological Chemistry*.

[33] Burgermeister E, Chuderland D, Hanoch T, Meyer M, Liscovitch M, Seger R (2007). Interaction with MEK causes nuclear export and downregulation of peroxisome proliferator-activated receptor *γ*. *Molecular and Cellular Biology*.

[34] Michalik L, Desvergne B, Wahli W (2004). Peroxisome-proliferator-activated receptors and cancers: complex stories. *Nature Reviews Cancer*.

[35] Grommes C, Landreth GE, Sastre M (2006). Inhibition of in vivo glioma growth and invasion by peroxisome proliferator-activated receptor *γ* agonist treatment. *Molecular Pharmacology*.

[36] Cui Y, Lu Z, Bai L, Shi Z, Zhao W-E, Zhao B (2007). *β*-carotene induces apoptosis and up-regulates peroxisome proliferator-activated receptor *γ* expression and reactive oxygen species production in MCF-7 cancer cells. *European Journal of Cancer*.

[37] Sun H, Berquin IM, Owens RT, O'Flaherty JT, Edwards IJ (2008). Peroxisome proliferator-activated receptor *β*-mediated up-regulation of syndecan-1 by n-3 fatty acids promotes apoptosis of human breast cancer cells. *Cancer Research*.

[38] Borbath I, Leclercq I, Moulin P, Sempoux C, Horsmans Y (2007). The PPARgamma agonist pioglitazone inhibits early neoplastic occurrence in the rat liver. *European Journal of Cancer*.

[39] Folkman J (2007). Angiogenesis: an organizing principle for drug discovery?. *Nature Reviews Drug Discovery*.

[40] Giaginis C, Tsantili-Kakoulidou A, Theocharis S (2008). Peroxisome proliferator-activated receptor-*γ* ligands: potential pharmacological agents for targeting the angiogenesis signaling cascade in cancer. *PPAR Research*.

[41] Panigrahy D, Huang S, Kieran MW, Kaipainen A (2005). PPAR*γ* as a therapeutic target for tumor angiogenesis and metastasis. *Cancer Biology and Therapy*.

[42] Keshamouni VG, Arenberg DA, Reddy RC, Newstead MJ, Anthwal S, Standiford TJ (2005). PPAR-*γ* activation inhibits angiogenesis by blocking ELR+CXC chemokine production in non-small cell lung cancer. *Neoplasia*.

[43] Huang H, Campbell SC, Bedford DF (2004). Peroxisome proliferator-activated receptor *γ* ligands improve the antitumor efficacy of thrombospondin peptide ABT510. *Molecular Cancer Research*.

[44] McCarty MF, Barroso-Aranda J, Contreras F (2008). PPARgamma agonists can be expected to potentiate the efficacy of metronomic chemotherapy through CD36 up-regulation. *Medical Hypotheses*.

[45] Katoh M, Katoh M (2007). WNT signaling pathway and stem cell signaling network. *Clinical Cancer Research*.

[46] Galli A, Mello T, Ceni E, Surrenti E, Surrenti C (2006). The potential of antidiabetic thiazolidinediones for anticancer therapy. *Expert Opinion on Investigational Drugs*.

[47] Yee LD, Williams N, Wen P (2007). Pilot study of rosiglitazone therapy in women with breast cancer: effects of short-term therapy on tumor tissue and serum markers. *Clinical Cancer Research*.

[48] Lefebvre A-M, Paulweber B, Fajas L (1999). Peroxisome proliferator-activated receptor gamma is induced during differentiation of colon epithelium cells. *Journal of Endocrinology*.

[49] Sarraf P, Mueller E, Jones D (1998). Differentiation and reversal of malignant changes in colon cancer through PPAR*γ*. *Nature Medicine*.

[50] Gupta RA, Sarraf P, Brockman JA (2003). Peroxisome proliferator-activated receptor *γ* and transforming growth factor-*β* pathways inhibit intestinal epithelial cell growth by regulating levels of TSC-22. *The Journal of Biological Chemistry*.

[51] Chang AJ, Song DH, Wolfe MM (2006). Attenuation of peroxisome proliferator-activated receptor *γ* (PPAR*γ*) mediates gastrin-stimulated colorectal cancer cell proliferation. *The Journal of Biological Chemistry*.

[52] Yamazaki K, Shimizu M, Okuno M (2007). Synergistic effects of RXR*α* and PPAR*γ* ligands to inhibit growth in human colon 
cancer cells—phosphorylated RXR*α* is a critical target for colon cancer management. *Gut*.

[53] Girnun GD, Smith WM, Drori S (2002). APC-dependent suppression of colon carcinogenesis by PPAR*γ*. *Proceedings of the National Academy of Sciences of the United States of America*.

[54] Saez E, Tontonoz P, Nelson MC (1998). Activators of the nuclear receptor PPAR*γ* enhance colon polyp formation. *Nature Medicine*.

[55] Niho N, Takahashi M, Shoji Y (2003). Dose-dependent suppression of hyperlipidemia and intestinal polyp formation in Min mice by pioglitazone, a PPAR*γ* ligand. *Cancer Science*.

[56] McAlpine CA, Barak Y, Matise I, Cormier RT (2006). Intestinal-specific PPAR*γ* deficiency enhances tumorigenesis in Apc^Min/+^ mice. *International Journal of Cancer*.

[57] Thompson EA (2007). PPAR*γ* physiology and pathology in gastrointestinal epithelial cells. *Molecules and Cells*.

[58] Kulke MH, Demetri GD, Sharpless NE (2002). A phase II study of troglitazone, an activator of the PPAR*γ* receptor, in patients with chemotherapy-resistant metastatic colorectal cancer. *Cancer Journal*.

[59] Bach PB, Silvestri GA, Hanger M, Jett JR (2007). Screening for lung cancer: ACCP evidence-based clinical practice guidelines (2nd edition). *Chest*.

[60] Soria J-C, Kim ES, Fayette J, Lantuejoul S, Deutsch E, Hong WK (2003). Chemoprevention of lung cancer. *The Lancet Oncology*.

[61] Hirsch FR, Lippman SM (2005). Advances in the biology of lung cancer chemoprevention. *Journal of Clinical Oncology*.

[62] Chang T-H, Szabo E (2000). Induction of differentiation and apoptosis by ligands of peroxisome proliferator-activated receptor *γ* in non-small cell lung cancer. *Cancer Research*.

[63] Tsubouchi Y, Sano H, Kawahito Y (2000). Inhibition of human lung cancer cell growth by the peroxisome proliferator-activated receptor-*γ* agonists through induction of apoptosis. *Biochemical and Biophysical Research Communications*.

[64] Wick M, Hurteau G, Dessev C (2002). Peroxisome proliferator-activated receptor-*γ* is a target of nonsteroidal anti-inflammatory drugs mediating cyclooxygenase-independent inhibition of lung cancer cell growth. *Molecular Pharmacology*.

[65] Lee SY, Hur GY, Jung KH (2006). PPAR-*γ* agonist increase gefitinib's antitumor activity through PTEN expression. *Lung Cancer*.

[66] Han S, Sidell N, Fisher PB, Roman J (2004). Up-regulation of p21 gene expression by peroxisome proliferator-activated 
receptor *γ* in human lung carcinoma 
cells. *Clinical Cancer Research*.

[67] Keshamouni VG, Reddy RC, Arenberg DA (2004). Peroxisome proliferator-activated receptor-*γ* activation inhibits tumor progression in non-small-cell lung cancer. *Oncogene*.

[68] Han S, Rivera HN, Roman J (2005). Peroxisome proliferator-activated receptor-*γ* ligands inhibit *α*5 integrin gene transcription in non-small cell lung carcinoma cells. *American Journal of Respiratory Cell and Molecular Biology*.

[69] Govindarajan R, Ratnasinghe L, Simmons DL (2007). Thiazolidinediones and the risk of lung, prostate, and colon cancer in patients with diabetes. *Journal of Clinical Oncology*.

[70] Nissen SE, Wolski K (2007). Effect of rosiglitazone on the risk of myocardial infarction and death from cardiovascular causes. *The New England Journal of Medicine*.

[71] Nemenoff RA (2007). Peroxisome proliferator-activated receptor-*γ* in lung 
cancer: defining specific versus “off-target” effectors. *Journal of Thoracic Oncology*.

[72] Hazra S, Batra RK, Tai HH, Sharma S, Cui X, Dubinett SM (2007). Pioglitazone and rosiglitazone decrease prostaglandin E_2_ in non-small-cell lung cancer cells by up-regulating 15-hydroxyprostaglandin dehydrogenase. *Molecular Pharmacology*.

[73] Zou W, Liu X, Yue P, Khuri FR, Sun S-Y (2007). PPAR*γ* ligands enhance TRAIL-induced apoptosis through DR5 upregulation and c-FLIP downregulation in human lung cancer cells. *Cancer Biology and Therapy*.

[74] Yao C-J, Lai G-M, Chan C-F, Cheng A-L, Yang Y-Y, Chuang S-E (2006). Dramatic synergistic anticancer effect of clinically achievable doses of lovastatin and troglitazone. *International Journal of Cancer*.

[75] Avis I, Martínez A, Tauler J (2005). Inhibitors of the arachidonic acid pathway and peroxisome proliferator-activated receptor ligands have superadditive effects on lung cancer growth inhibition. *Cancer Research*.

[76] Dorgan JF, Sowell A, Swanson CA (1998). Relationships of serum carotenoids, retinol, *α*-tocopherol, and selenium with breast cancer 
risk: results from a prospective study in Columbia, Missouri (United States). *Cancer Causes & Control*.

[77] Toniolo P, Van Kappel AL, Akhmedkhanov A (2001). Serum carotenoids and breast cancer. *American Journal of Epidemiology*.

[78] Mueller E, Sarraf P, Tontonoz P (1998). Terminal differentiation of human breast cancer through PPAR*γ*. *Molecular Cell*.

[79] Mehta RG, Williamson E, Patel MK, Koeffler HP (2000). A ligand of peroxisome proliferator-activated receptor *γ*, retinoids, and prevention of preneoplastic mammary lesions. *Journal of the National Cancer Institute*.

[80] Elstner E, Müller C, Koshizuka K (1998). Ligands for peroxisome proliferator-activated receptory and retinoic acid receptor inhibit growth and induce apoptosis of human breast cancer cells in vitro and in BNX mice. *Proceedings of the National Academy of Sciences of the United States of America*.

[81] Burstein HJ, Demetri GD, Mueller E, Sarraf P, Spiegelman BM, Winer EP (2003). Use of the peroxisome proliferator-activated receptor (PPAR) *γ* ligand troglitazone as treatment 
for refractory breast cancer: a phase II study. *Breast Cancer Research and Treatment*.

[82] Mueller E, Smith M, Sarraf P (2000). Effects of ligand activation of peroxisome proliferator-activated receptor *γ* in human prostate cancer. *Proceedings of the National Academy of Sciences of the United States of America*.

[83] Sabichi AL, Subbarayan V, Llansa N, Lippman SM, Menter DG (2004). Peroxisome proliferator-activated receptor-*γ* suppresses cyclooxygenase-2 expression in human prostate cells. *Cancer Epidemiology Biomarkers & Prevention*.

[84] Shiau C-W, Yang C-C, Kulp SK (2005). Thiazolidenediones mediate apoptosis in prostate cancer cells in part through inhibition of Bcl-xL/Bcl-2 functions independently of PPAR*γ*. *Cancer Research*.

[85] Hisatake J-I, Ikezoe T, Carey M, Holden S, Tomoyasu S, Koeffler HP (2000). Down-regulation of prostate-specific antigen expression by ligands for peroxisome proliferator-activated receptor *γ* in human prostate cancer. *Cancer Research*.

[86] Wu W, Celestino J, Milam MR (2008). Primary chemoprevention of endometrial hyperplasia with the peroxisome 
proliferator-activated receptor gamma agonist rosiglitazone in the *PTEN* heterozygote murine model. *International Journal of Gynecological Cancer*.

[87] Elnemr A, Ohta T, Iwata K (2000). PPARgamma ligand (thiazolidinedione) induces growth arrest and differentiation markers of human pancreatic cancer cells. *International Journal of Oncology*.

[88] Motomura W, Okumura T, Takahashi N, Obara T, Kohgo Y (2000). Activation of peroxisome proliferator-activated receptor *γ* by troglitazone inhibits cell growth through the increase of p27^Kip1^ in human pancreatic carcinoma cells. *Cancer Research*.

[89] Toyota M, Miyazaki Y, Kitamura S (2002). Peroxisome proliferator-activated receptor *γ* reduces the growth rate of pancreatic cancer cells through the reduction of cyclin D1. *Life Sciences*.

[90] Tontonoz P, Singer S, Forman BM (1997). Terminal differentiation of human liposarcoma cells induced by ligands for peroxisome proliferator-activated receptor *γ* and the retinoid X receptor. *Proceedings of the National Academy of Sciences of the United States of America*.

[91] Debrock G, Vanhentenrijk V, Sciot R, Debiec-Rychter M, Oyen R, Van Oosterom A (2003). A phase II trial with rosiglitazone in liposarcoma patients. *British Journal of Cancer*.

[92] Kroll TG, Sarraf P, Pecciarini L (2000). PAX8-PPAR*γ*1 fusion in oncogene human thyroid carcinoma. *Science*.

[93] Shen WT, Chung W-Y (2005). Treatment of thyroid cancer with histone deacetylase inhibitors and peroxisome proliferator-activated receptor-*γ* agonists. *Thyroid*.

[94] Klopper JP, Hays WR, Sharma V, Baumbusch MA, Hershman JM, Haugen BR (2004). Retinoid X receptor-*γ* and peroxisome proliferator-activated receptor-*γ* expression predicts thyroid carcinoma cell response to retinoid and thiazolidinedione treatment. *Molecular Cancer Therapeutics*.

[95] Fabiani R, De Bartolomeo A, Rosignoli P, Servili M, Montedoro GF, Morozzi G (2002). Cancer chemoprevention by hydroxytyrosol isolated from virgin olive oil through G1 
cell cycle arrest and apoptosis. *European Journal of Cancer Prevention*.

[96] D'Incalci M, Steward WP, Gescher AJ (2005). Use of cancer chemopreventive phytochemicals as antineoplastic agents. *The Lancet Oncology*.

[97] Lagiou P, Rossi M, Lagiou A, Tzonou A, La Vecchia C, Trichopoulos D (2008). Flavonoid intake and liver cancer: a case-control study in Greece. *Cancer Causes & Control*.

[98] Linseisen J, Rohrmann S, Miller AB (2007). Fruit and vegetable consumption and lung cancer risk: updated information from 
the European Prospective Investigation into Cancer and Nutrition (EPIC). *International Journal of Cancer*.

[99] Ko JKS, Leung WC, Ho WK, Chiu P (2007). Herbal diterpenoids induce growth arrest and apoptosis in colon cancer cells with increased expression of the nonsteroidal anti-inflammatory drug-activated gene. *European Journal of Pharmacology*.

[100] Shishodia S, Sethi G, Konopleva M, Andreeff M, Aggarwal BB (2006). A synthetic triterpenoid, CDDO-Me, inhibits I*κ*B*α* kinase and enhances apoptosis induced by TNF and chemotherapeutic agents 
through down-regulation of expression of nuclear factor *κ*B-regulated gene products in human leukemic cells. *Clinical Cancer Research*.

[101] Amico V, Barresi V, Condorelli D, Spatafora C, Tringali C (2006). Antiproliferative terpenoids from almond hulls (*Prunus dulcis*): identification and 
structure-activity relationships. *Journal of Agricultural and Food Chemistry*.

[102] Hsieh C-L, Tseng M-H, Shao Y-Y (2006). C35 terpenoids from the bark of Calocedrus macrolepis var. formosana with activity 
against human cancer cell lines. *Journal of Natural Products*.

[103] Lapillonne H, Konopleva M, Tsao T (2003). Activation of peroxisome proliferator-activated receptor *γ* by a novel synthetic triterpenoid 2-cyano-3,12-dioxooleana-1,9-dien-28-oic acid induces growth arrest and 
apoptosis in breast cancer cells. *Cancer Research*.

[104] Chintharlapalli S, Papineni S, Jutooru I, McAlees A, Safe S (2007). Structure-dependent activity of glycyrrhetinic acid derivatives as peroxisome proliferator-activated receptor *γ* agonists in colon 
cancer cells. *Molecular Cancer Therapeutics*.

[105] Chintharlapalli S, Papineni S, Liu S (2007). 2-cyano-lup-1-en-3-oxo-20-oic acid, a cyano derivative of betulinic acid, activates peroxisome proliferator-activated receptor *γ* in colon and pancreatic cancer cells. *Carcinogenesis*.

[106] Ho C-C, Huang P-H, Huang H-Y, Chen Y-H, Yang P-C, Hsu S-M (2002). Up-regulated caveolin-1 accentuates the metastasis capability of lung adenocarcinoma by inducing filopodia formation. *American Journal of Pathology*.

[107] Burgermeister E, Tencer L, Liscovitch M (2003). Peroxisome proliferator-activated receptor-*γ* upregulates caveolin-1 and caveolin-2 expression in human carcinoma cells. *Oncogene*.

[108] Ricketts M-L, Moore DD, Banz WJ, Mezei O, Shay NF (2005). Molecular mechanisms of action of the soy isoflavones includes activation of promiscuous nuclear receptors. A review. *The Journal of Nutritional Biochemistry*.

[109] Dang Z-C, Audinot V, Papapoulos SE, Boutin JA, Löwik CWGM (2003). Peroxisome proliferator-activated receptor *γ* (PPAR*γ*) as a molecular target for the soy phytoestrogen genistein. *The Journal of Biological Chemistry*.

[110] Liang Y-C, Tsai S-H, Tsai D-C, Lin-Shiau S-Y, Lin J-K (2001). Suppression of inducible cyclooxygenase and nitric oxide synthase through activation of peroxisome proliferator-activated receptor-*γ* by flavonoids in mouse macrophages. *FEBS Letters*.

[111] Fischer L, Mahoney C, Jeffcoat AR (2004). Clinical characteristics and pharmacokinetics of purified soy isoflavones: multiple-dose 
administration to men with prostate neoplasia. *Nutrition and Cancer*.

[112] Kumar NB, Krischer JP, Allen K (2007). Safety of purified isoflavones in men with clinically localized prostate cancer. *Nutrition and Cancer*.

[113] Kumar NB, Krischer JP, Allen K (2007). A phase II randomized, placebo-controlled clinical trial of purified isoflavones in modulating steroid hormones in men diagnosed with localized prostate cancer. *Nutrition and Cancer*.

[114] Takimoto CH, Glover K, Huang X (2003). Phase I pharmacokinetic and pharmacodynamic analysis of unconjugated soy isoflavones administered to individuals with cancer. *Cancer Epidemiology Biomarkers & Prevention*.

[115] Hosokawa M, Kudo M, Maeda H, Kohno H, Tanaka T, Miyashita K (2004). Fucoxanthin induces apoptosis and enhances the antiproliferative effect of the PPAR*γ* ligand, troglitazone, on colon cancer cells. *Biochimica et Biophysica Acta*.

[116] Maggiora M, Bologna M, Cerù MP (2004). An overview of the effect of linoleic and conjugated-linoleic acids on the growth of several human tumor cell lines. *International Journal of Cancer*.

[117] Bocca C, Bozzo F, Francica S, Colombatto S, Miglietta A (2007). Involvement of PPAR*γ* and E-cadherin/*β*-catenin pathway in the antiproliferative effect of conjugated linoleic acid in MCF-7 cells. *International Journal of Cancer*.

[118] Tsuzuki T, Kawakami Y (2008). Tumor angiogenesis suppression by *α*-eleostearic acid, a linolenic acid isomer with a conjugated triene system, via peroxisome proliferator-activated receptor *γ*. *Carcinogenesis*.

[119] Yasui Y, Suzuki R, Kohno H (2007). 9trans,11trans conjugated linoleic acid inhibits the development of azoxymethane-induced colonic aberrant crypt foci in rats. *Nutrition and Cancer*.

[120] Sasaki T, Yoshida K, Shimura H (2006). Inhibitory effect of linoleic acid on transformation of IEC6 intestinal cells by in vitro azoxymethane treatment. *International Journal of Cancer*.

[121] Bull AW, Steffensen KR, Leers J, Rafter JJ (2003). Activation of PPAR *γ* in colon tumor cell lines by oxidized metabolites of linoleic acid, endogenous ligands for PPAR *γ*. *Carcinogenesis*.

[122] Zuo X, Wu Y, Morris JS (2006). Oxidative metabolism of linoleic acid modulates PPAR-beta/delta 
suppression of PPAR-gamma activity. *Oncogene*.

[123] Kim C-S, Park W-H, Park J-Y (2004). Capsaicin, a spicy component of hot pepper, induces apoptosis by activation of the peroxisome proliferator-activated receptor *γ* in HT-29 human colon cancer cells. *Journal of Medicinal Food*.

[124] Mori A, Lehmann S, O'Kelly J (2006). Capsaicin, a component of red peppers, inhibits the growth of androgen-independent, p53 mutant prostate cancer cells. *Cancer Research*.

[125] Jun H-S, Park T, Lee CK (2007). Capsaicin induced apoptosis of B16-F10 melanoma cells through down-regulation of Bcl-2. *Food and Chemical Toxicology*.

[126] Surh Y-J, Lee SS (1996). Capsaicin in hot chili pepper: carcinogen, co-carcinogen or anticarcinogen?. *Food and Chemical Toxicology*.

[127] Yoshitani SI, Tanaka T, Kohno H, Takashima S (2001). Chemoprevention of azoxymethane-induced rat colon carcinogenesis by dietary capsaicin and rotenone. *International Journal of Oncology*.

[128] Clapper ML, Szarka CE, Pfeiffer GR (1997). Preclinical and clinical evaluation of broccoli supplements as inducers of 
glutathione *S*-transferase activity. *Clinical Cancer Research*.

[129] Sharma RA, Ireson CR, Verschoyle RD (2001). Effects of dietary curcumin on glutathione *S*-transferase and malondialdehyde-DNA adducts in rat liver and colon 
mucosa: relationship with drug levels. *Clinical Cancer Research*.

[130] Park EY, Cho IJ, Kim SG (2004). Transactivation of the PPAR-responsive enhancer module in chemopreventive 
glutathione *S*-transferase gene by the peroxisome proliferator-activated receptor-*γ* and retinoid X receptor heterodimer. *Cancer Research*.

